# (*O*-Methyl di­thio­carbonato-κ*S*)tri­phenyl­tin(IV)

**DOI:** 10.1107/S1600536813012300

**Published:** 2013-05-15

**Authors:** Fatima Javed, Saqib Ali, Wajid Shah, M. Nawaz Tahir, Hameed Ullah

**Affiliations:** a Department of Chemistry, Quaid-i-Azam University, Islamabad, Pakistan; b Department of Chemistry, University of Hazara, K.P.K, Pakistan; c Department of Physics, University of Sargodha, Sargodha, Pakistan; d Department of Chemistry, Hazara University, Mansehra, Pakistan

## Abstract

In the title compound, [Sn(C_6_H_5_)_3_(C_2_H_3_OS_2_)], the Sn^IV^ atom adopts a distorted SnC_3_S tetra­hedral coordination geometry. A short Sn⋯O contact [2.988 (4) Å] is also present. The phenyl rings are each disordered over two sets of sites with an occupancy ratio of 0.550 (8):0.450 (8). The crystal studied was found to be a racemic twin with a twin component ratio of 0.57 (18):0.43 (18).

## Related literature

For a related structure, see: Ng & Rae (2000 ▶).
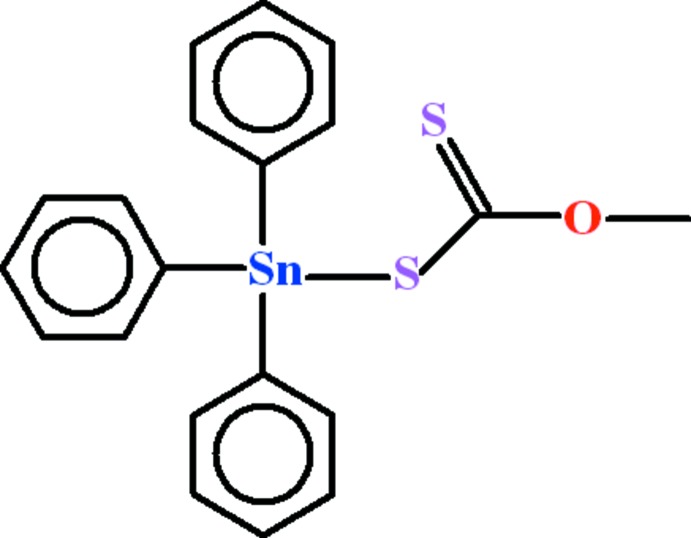


## Experimental

### 

#### Crystal data

[Sn(C_6_H_5_)_3_(C_2_H_3_OS_2_)]
*M* *_r_* = 457.15
Monoclinic, 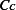

*a* = 17.4400 (9) Å
*b* = 8.4734 (4) Å
*c* = 15.6069 (9) Å
β = 119.626 (2)°
*V* = 2004.82 (19) Å^3^
*Z* = 4
Mo *K*α radiation
μ = 1.49 mm^−1^
*T* = 296 K
0.28 × 0.22 × 0.18 mm

#### Data collection

Bruker Kappa APEXII CCD diffractometer
Absorption correction: multi-scan (*SADABS*; Bruker, 2005 ▶) *T*_min_ = 0.683, *T*_max_ = 0.763
7166 measured reflections
3483 independent reflections
3341 reflections with *I* > 2σ(*I*)
*R*_int_ = 0.014

#### Refinement

*R*[*F* ^2^ > 2σ(*F* ^2^)] = 0.022
*wR*(*F* ^2^) = 0.055
*S* = 1.04
3483 reflections
112 parameters
2 restraints
H-atom parameters constrained
Δρ_max_ = 0.44 e Å^−3^
Δρ_min_ = −0.29 e Å^−3^
Absolute structure: Flack (1983 ▶), 1387 Friedel pairs
Flack parameter: 0.43 (18)

### 

Data collection: *APEX2* (Bruker, 2007 ▶); cell refinement: *SAINT* (Bruker, 2007 ▶); data reduction: *SAINT*; program(s) used to solve structure: *SHELXS97* (Sheldrick, 2008 ▶); program(s) used to refine structure: *SHELXL97* (Sheldrick, 2008 ▶); molecular graphics: *ORTEP-3 for Windows* (Farrugia, 2012 ▶) and *PLATON* (Spek, 2009 ▶); software used to prepare material for publication: *WinGX* (Farrugia, 2012 ▶) and *PLATON*.

## Supplementary Material

Crystal structure: contains datablock(s) global, I. DOI: 10.1107/S1600536813012300/hb7077sup1.cif

Structure factors: contains datablock(s) I. DOI: 10.1107/S1600536813012300/hb7077Isup2.hkl

Additional supplementary materials:  crystallographic information; 3D view; checkCIF report

## Figures and Tables

**Fig. 1.**
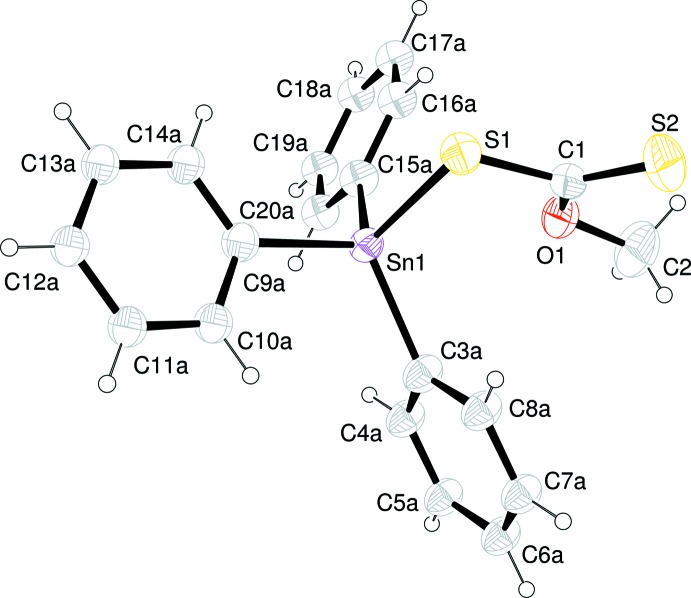
View of the title compound with displacement ellipsoids drawn at the 50% probability level. H-atoms are shown by small circles of arbitrary radii. Only the major part of atoms of disordered group are shown for clarity.

## supplementary crystallographic information

### Comment

The crystal structure of (*O*-methyl dithiocarbonato-*S*)-triphenyl-tin(iv) (Ng & Rae, 2000) has been reported, which is related to title compound (I).

In (I) the coordination around tin atom is distorted tetrahedral with three C-atoms and an S-atom. The tin and coordinated sulpher atom is at a distance of -0.5497 (47) Å and -2.9805 (53) Å from the plane (C3A/C9A/C15A). The phenyl rings are disordered over two sites with occupancy ratio of 0.550 (8):0.450 (8). The dihedral angle between disordered rings (C3A–C8A)/(C3B–C8B), (C9A–C14A)/(C9B–C14B) and (C15A–C20A)/(C15B–C20B) is 19.2 (7)°, 8.3 (10)° and 14.1 (5)°, respectively. The *O*-methyl hydrogen carbonodithioate moiety (S1/C1/S2/O1/C2) is planar with r. m. s. deviation of 0.0326 Å and is oriented at a dihedral angle of 71.22 (19)° with the plane (C3A/C9A/C15A).

### Experimental

Potassium-*O*-methyl carrbonodithioate (10 mmol, 1.46 g) was dissolved in methanol and stirred for 30 min. Then triphenytin(IV) chloride was added dropwise and the reaction mixture was stirred for 7–8 h till white precipitates were appeared. The resultant mixture was filtered to remove KCl salt. The filtrate was rotary evaporated to get white solid mass and recrystalized from chloroform to obtaine colourless prisms of (I) in 85.33% yield.

### Refinement

There is a recamic twin with BASF value of 0.43147.

The benzene rings of triphenyl tin are disordered over two sites with occupancy ratio of 0.550 (8):0.450 (8) and were idealized as regular hexagons with C–C bond distance equal to 1.39 Å. The C-atoms of respective rings were treated as anisotropic with equal thermal parameters.

The H-atoms were positioned geometrically (C–H = 0.93–0.96 Å) and refined as riding with *U*_iso_(H) = *xU*_eq_(C), where *x* = 1.5 for methyl and *x* = 1.2 for other H-atoms.

### Crystal data

**Table:**

[Sn(C_6_H_5_)_3_(C_2_H_3_OS_2_)]	*F*(000) = 912
*M**_r_* = 457.15	*D*_x_ = 1.515 Mg m^−^^3^
Monoclinic, *C**c*	Mo *K*α radiation, λ = 0.71073 Å
Hall symbol: C -2yc	Cell parameters from 3341 reflections
*a* = 17.4400 (9) Å	θ = 2.8–26.0°
*b* = 8.4734 (4) Å	µ = 1.49 mm^−^^1^
*c* = 15.6069 (9) Å	*T* = 296 K
β = 119.626 (2)°	Prism, white
*V* = 2004.82 (19) Å^3^	0.28 × 0.22 × 0.18 mm
*Z* = 4	

### Data collection

**Table:**

Bruker Kappa APEXII CCD diffractometer	3483 independent reflections
Radiation source: fine-focus sealed tube	3341 reflections with *I* > 2σ(*I*)
Graphite monochromator	*R*_int_ = 0.014
Detector resolution: 8.00 pixels mm^-1^	θ_max_ = 26.0°, θ_min_ = 2.8°
ω scans	*h* = −21→20
Absorption correction: multi-scan (*SADABS*; Bruker, 2005)	*k* = −10→10
*T*_min_ = 0.683, *T*_max_ = 0.763	*l* = −19→19
7166 measured reflections	

### Refinement

**Table:**

Refinement on *F*^2^	Secondary atom site location: difference Fourier map
Least-squares matrix: full	Hydrogen site location: inferred from neighbouring sites
*R*[*F*^2^ > 2σ(*F*^2^)] = 0.022	H-atom parameters constrained
*wR*(*F*^2^) = 0.055	*w* = 1/[σ^2^(*F*_o_^2^) + (0.0246*P*)^2^ + 2.1104*P*] where *P* = (*F*_o_^2^ + 2*F*_c_^2^)/3
*S* = 1.04	(Δ/σ)_max_ < 0.001
3483 reflections	Δρ_max_ = 0.44 e Å^−^^3^
112 parameters	Δρ_min_ = −0.29 e Å^−^^3^
2 restraints	Absolute structure: Flack (1983), 1387 Friedel pairs
Primary atom site location: structure-invariant direct methods	Flack parameter: 0.43 (18)

### Special details

**Table:**

Geometry. Bond distances, angles *etc*. have been calculated using the rounded fractional coordinates. All su's are estimated from the variances of the (full) variance-covariance matrix. The cell e.s.d.'s are taken into account in the estimation of distances, angles and torsion angles
Refinement. Refinement of *F*^2^ against ALL reflections. The weighted *R*-factor *wR* and goodness of fit *S* are based on *F*^2^, conventional *R*-factors *R* are based on *F*, with *F* set to zero for negative *F*^2^. The threshold expression of *F*^2^ > σ(*F*^2^) is used only for calculating *R*-factors(gt) *etc*. and is not relevant to the choice of reflections for refinement. *R*-factors based on *F*^2^ are statistically about twice as large as those based on *F*, and *R*- factors based on ALL data will be even larger.

### Fractional atomic coordinates and isotropic or equivalent isotropic displacement parameters (Å^2^)

**Table:**

	*x*	*y*	*z*	*U*_iso_*/*U*_eq_	Occ. (<1)
Sn1	0.01045 (1)	0.18518 (2)	0.25080 (1)	0.0459 (1)	
S1	−0.10922 (8)	0.30516 (13)	0.10239 (9)	0.0611 (4)	
S2	−0.11228 (15)	0.6208 (2)	0.02348 (15)	0.1128 (7)	
O1	0.0289 (2)	0.4577 (4)	0.1408 (3)	0.0687 (11)	
C1	−0.0567 (3)	0.4711 (5)	0.0901 (3)	0.0581 (14)	
C2	0.0828 (4)	0.5909 (7)	0.1467 (5)	0.106 (3)	
C3A	0.0722 (5)	0.3677 (6)	0.3576 (5)	0.0589 (8)	0.550 (8)
C4A	0.1594 (5)	0.3461 (6)	0.4305 (5)	0.0589 (8)	0.550 (8)
C5A	0.2039 (4)	0.4656 (8)	0.4982 (5)	0.0589 (8)	0.550 (8)
C6A	0.1611 (4)	0.6067 (6)	0.4930 (4)	0.0589 (8)	0.550 (8)
C7A	0.0738 (4)	0.6283 (6)	0.4200 (5)	0.0589 (8)	0.550 (8)
C8A	0.0294 (4)	0.5088 (8)	0.3523 (5)	0.0589 (8)	0.550 (8)
C9A	−0.0659 (5)	0.0278 (10)	0.2859 (7)	0.0590 (9)	0.550 (8)
C10A	−0.0786 (6)	0.0702 (8)	0.3640 (6)	0.0590 (9)	0.550 (8)
C11A	−0.1305 (5)	−0.0234 (8)	0.3883 (5)	0.0590 (9)	0.550 (8)
C12A	−0.1697 (4)	−0.1594 (7)	0.3345 (5)	0.0590 (9)	0.550 (8)
C13A	−0.1570 (4)	−0.2018 (7)	0.2564 (5)	0.0590 (9)	0.550 (8)
C14A	−0.1051 (5)	−0.1081 (10)	0.2320 (6)	0.0590 (9)	0.550 (8)
C15A	0.0947 (4)	0.0647 (8)	0.2102 (5)	0.0576 (8)	0.550 (8)
C16A	0.0687 (3)	0.0619 (8)	0.1104 (5)	0.0576 (8)	0.550 (8)
C17A	0.1207 (4)	−0.0140 (7)	0.0785 (4)	0.0576 (8)	0.550 (8)
C18A	0.1987 (3)	−0.0872 (7)	0.1464 (5)	0.0576 (8)	0.550 (8)
C19A	0.2247 (3)	−0.0844 (8)	0.2462 (4)	0.0576 (8)	0.550 (8)
C20A	0.1727 (4)	−0.0084 (8)	0.2780 (4)	0.0576 (8)	0.550 (8)
C16B	0.0959 (5)	0.0687 (10)	0.1263 (6)	0.0576 (8)	0.450 (8)
C17B	0.1575 (6)	−0.0144 (9)	0.1123 (6)	0.0576 (8)	0.450 (8)
C18B	0.2203 (5)	−0.1074 (9)	0.1878 (7)	0.0576 (8)	0.450 (8)
C19B	0.2215 (4)	−0.1172 (9)	0.2773 (6)	0.0576 (8)	0.450 (8)
C20B	0.1598 (5)	−0.0341 (10)	0.2913 (5)	0.0576 (8)	0.450 (8)
C5B	0.1948 (4)	0.5125 (9)	0.4774 (7)	0.0589 (8)	0.450 (8)
C6B	0.1388 (5)	0.6235 (7)	0.4831 (6)	0.0589 (8)	0.450 (8)
C7B	0.0479 (5)	0.6058 (9)	0.4259 (6)	0.0589 (8)	0.450 (8)
C8B	0.0130 (4)	0.4772 (10)	0.3630 (7)	0.0589 (8)	0.450 (8)
C9B	−0.0674 (6)	0.0296 (11)	0.2813 (9)	0.0590 (9)	0.450 (8)
C10B	−0.0998 (6)	0.0581 (9)	0.3453 (7)	0.0590 (9)	0.450 (8)
C11B	−0.1561 (5)	−0.0502 (9)	0.3528 (6)	0.0590 (9)	0.450 (8)
C12B	−0.1800 (5)	−0.1870 (8)	0.2963 (6)	0.0590 (9)	0.450 (8)
C13B	−0.1476 (6)	−0.2155 (9)	0.2323 (6)	0.0590 (9)	0.450 (8)
C14B	−0.0913 (7)	−0.1072 (12)	0.2248 (8)	0.0590 (9)	0.450 (8)
C15B	0.0970 (5)	0.0588 (10)	0.2158 (7)	0.0576 (8)	0.450 (8)
C4B	0.1599 (6)	0.3839 (8)	0.4144 (7)	0.0589 (8)	0.450 (8)
C3B	0.0690 (6)	0.3662 (8)	0.3572 (6)	0.0589 (8)	0.450 (8)
H20A	0.19005	−0.00656	0.34478	0.0691*	0.550 (8)
H19A	0.27690	−0.13333	0.29154	0.0691*	0.550 (8)
H6A	0.19081	0.68660	0.53825	0.0706*	0.550 (8)
H7A	0.04520	0.72266	0.41647	0.0706*	0.550 (8)
H8A	−0.02897	0.52327	0.30348	0.0706*	0.550 (8)
H10A	−0.05241	0.16116	0.39997	0.0708*	0.550 (8)
H11A	−0.13903	0.00491	0.44055	0.0708*	0.550 (8)
H12A	−0.20441	−0.22204	0.35075	0.0708*	0.550 (8)
H13A	−0.18317	−0.29273	0.22036	0.0708*	0.550 (8)
H14A	−0.09654	−0.13648	0.17978	0.0708*	0.550 (8)
H16A	0.01651	0.11088	0.06504	0.0691*	0.550 (8)
H17A	0.10335	−0.01589	0.01181	0.0691*	0.550 (8)
H18A	0.23355	−0.13799	0.12506	0.0691*	0.550 (8)
H2A	0.07169	0.61782	0.08181	0.1596*	
H2B	0.14393	0.56420	0.18742	0.1596*	
H2C	0.06866	0.67927	0.17483	0.1596*	
H4A	0.18807	0.25175	0.43404	0.0706*	0.550 (8)
H5A	0.26224	0.45114	0.54703	0.0706*	0.550 (8)
H4B	0.19733	0.30958	0.41060	0.0706*	0.450 (8)
H5B	0.25556	0.52423	0.51568	0.0706*	0.450 (8)
H6B	0.16213	0.70949	0.52526	0.0706*	0.450 (8)
H7B	0.01046	0.68010	0.42975	0.0706*	0.450 (8)
H8B	−0.04778	0.46545	0.32467	0.0706*	0.450 (8)
H10B	−0.08378	0.14963	0.38313	0.0708*	0.450 (8)
H11B	−0.17772	−0.03112	0.39561	0.0708*	0.450 (8)
H12B	−0.21762	−0.25945	0.30127	0.0708*	0.450 (8)
H13B	−0.16359	−0.30704	0.19445	0.0708*	0.450 (8)
H14B	−0.06966	−0.12630	0.18198	0.0708*	0.450 (8)
H16B	0.05384	0.13082	0.07576	0.0691*	0.450 (8)
H17B	0.15674	−0.00783	0.05235	0.0691*	0.450 (8)
H18B	0.26155	−0.16294	0.17837	0.0691*	0.450 (8)
H19B	0.26346	−0.17939	0.32780	0.0691*	0.450 (8)
H20B	0.16056	−0.04074	0.35121	0.0691*	0.450 (8)

### Atomic displacement parameters (Å^2^)

**Table:**

	*U*^11^	*U*^22^	*U*^33^	*U*^12^	*U*^13^	*U*^23^
Sn1	0.0445 (1)	0.0449 (1)	0.0519 (1)	−0.0026 (2)	0.0266 (1)	−0.0031 (2)
S1	0.0473 (6)	0.0687 (7)	0.0598 (7)	−0.0047 (5)	0.0208 (5)	0.0035 (5)
S2	0.1330 (16)	0.0839 (10)	0.0928 (11)	0.0212 (10)	0.0338 (11)	0.0371 (9)
O1	0.071 (2)	0.0609 (18)	0.078 (2)	−0.0144 (15)	0.0398 (18)	0.0013 (15)
C1	0.074 (3)	0.052 (2)	0.050 (2)	0.000 (2)	0.032 (2)	−0.0010 (17)
C2	0.104 (4)	0.085 (4)	0.144 (5)	−0.036 (3)	0.072 (4)	−0.005 (4)
C3A	0.0513 (14)	0.0497 (13)	0.0659 (14)	0.0040 (10)	0.0216 (12)	−0.0055 (10)
C4A	0.0513 (14)	0.0497 (13)	0.0659 (14)	0.0040 (10)	0.0216 (12)	−0.0055 (10)
C5A	0.0513 (14)	0.0497 (13)	0.0659 (14)	0.0040 (10)	0.0216 (12)	−0.0055 (10)
C6A	0.0513 (14)	0.0497 (13)	0.0659 (14)	0.0040 (10)	0.0216 (12)	−0.0055 (10)
C7A	0.0513 (14)	0.0497 (13)	0.0659 (14)	0.0040 (10)	0.0216 (12)	−0.0055 (10)
C8A	0.0513 (14)	0.0497 (13)	0.0659 (14)	0.0040 (10)	0.0216 (12)	−0.0055 (10)
C9A	0.0570 (14)	0.0649 (12)	0.0597 (18)	−0.0151 (10)	0.0324 (12)	−0.0040 (11)
C10A	0.0570 (14)	0.0649 (12)	0.0597 (18)	−0.0151 (10)	0.0324 (12)	−0.0040 (11)
C11A	0.0570 (14)	0.0649 (12)	0.0597 (18)	−0.0151 (10)	0.0324 (12)	−0.0040 (11)
C12A	0.0570 (14)	0.0649 (12)	0.0597 (18)	−0.0151 (10)	0.0324 (12)	−0.0040 (11)
C13A	0.0570 (14)	0.0649 (12)	0.0597 (18)	−0.0151 (10)	0.0324 (12)	−0.0040 (11)
C14A	0.0570 (14)	0.0649 (12)	0.0597 (18)	−0.0151 (10)	0.0324 (12)	−0.0040 (11)
C15A	0.0493 (13)	0.0652 (13)	0.0660 (17)	−0.0020 (11)	0.0344 (13)	−0.0058 (12)
C16A	0.0493 (13)	0.0652 (13)	0.0660 (17)	−0.0020 (11)	0.0344 (13)	−0.0058 (12)
C17A	0.0493 (13)	0.0652 (13)	0.0660 (17)	−0.0020 (11)	0.0344 (13)	−0.0058 (12)
C18A	0.0493 (13)	0.0652 (13)	0.0660 (17)	−0.0020 (11)	0.0344 (13)	−0.0058 (12)
C19A	0.0493 (13)	0.0652 (13)	0.0660 (17)	−0.0020 (11)	0.0344 (13)	−0.0058 (12)
C20A	0.0493 (13)	0.0652 (13)	0.0660 (17)	−0.0020 (11)	0.0344 (13)	−0.0058 (12)
C16B	0.0493 (13)	0.0652 (13)	0.0660 (17)	−0.0020 (11)	0.0344 (13)	−0.0058 (12)
C17B	0.0493 (13)	0.0652 (13)	0.0660 (17)	−0.0020 (11)	0.0344 (13)	−0.0058 (12)
C18B	0.0493 (13)	0.0652 (13)	0.0660 (17)	−0.0020 (11)	0.0344 (13)	−0.0058 (12)
C19B	0.0493 (13)	0.0652 (13)	0.0660 (17)	−0.0020 (11)	0.0344 (13)	−0.0058 (12)
C20B	0.0493 (13)	0.0652 (13)	0.0660 (17)	−0.0020 (11)	0.0344 (13)	−0.0058 (12)
C5B	0.0513 (14)	0.0497 (13)	0.0659 (14)	0.0040 (10)	0.0216 (12)	−0.0055 (10)
C6B	0.0513 (14)	0.0497 (13)	0.0659 (14)	0.0040 (10)	0.0216 (12)	−0.0055 (10)
C7B	0.0513 (14)	0.0497 (13)	0.0659 (14)	0.0040 (10)	0.0216 (12)	−0.0055 (10)
C8B	0.0513 (14)	0.0497 (13)	0.0659 (14)	0.0040 (10)	0.0216 (12)	−0.0055 (10)
C9B	0.0570 (14)	0.0649 (12)	0.0597 (18)	−0.0151 (10)	0.0324 (12)	−0.0040 (11)
C10B	0.0570 (14)	0.0649 (12)	0.0597 (18)	−0.0151 (10)	0.0324 (12)	−0.0040 (11)
C11B	0.0570 (14)	0.0649 (12)	0.0597 (18)	−0.0151 (10)	0.0324 (12)	−0.0040 (11)
C12B	0.0570 (14)	0.0649 (12)	0.0597 (18)	−0.0151 (10)	0.0324 (12)	−0.0040 (11)
C13B	0.0570 (14)	0.0649 (12)	0.0597 (18)	−0.0151 (10)	0.0324 (12)	−0.0040 (11)
C14B	0.0570 (14)	0.0649 (12)	0.0597 (18)	−0.0151 (10)	0.0324 (12)	−0.0040 (11)
C15B	0.0493 (13)	0.0652 (13)	0.0660 (17)	−0.0020 (11)	0.0344 (13)	−0.0058 (12)
C4B	0.0513 (14)	0.0497 (13)	0.0659 (14)	0.0040 (10)	0.0216 (12)	−0.0055 (10)
C3B	0.0513 (14)	0.0497 (13)	0.0659 (14)	0.0040 (10)	0.0216 (12)	−0.0055 (10)

### Geometric parameters (Å, º)

**Table:**

Sn1—S1	2.4444 (13)	C16B—C17B	1.389 (14)
Sn1—C3A	2.134 (6)	C17A—C18A	1.390 (9)
Sn1—C9A	2.138 (10)	C17B—C18B	1.390 (12)
Sn1—C15A	2.127 (8)	C18A—C19A	1.391 (9)
Sn1—C3B	2.116 (8)	C18B—C19B	1.389 (13)
Sn1—C9B	2.109 (11)	C19A—C20A	1.389 (10)
Sn1—C15B	2.130 (10)	C19B—C20B	1.391 (12)
S1—C1	1.740 (5)	C2—H2A	0.9600
S2—C1	1.623 (5)	C2—H2B	0.9600
O1—C1	1.304 (7)	C2—H2C	0.9600
O1—C2	1.442 (8)	C4A—H4A	0.9300
C3A—C4A	1.389 (12)	C4B—H4B	0.9300
C3A—C8A	1.390 (10)	C5A—H5A	0.9300
C3B—C4B	1.391 (15)	C5B—H5B	0.9300
C3B—C8B	1.391 (13)	C6A—H6A	0.9300
C4A—C5A	1.390 (9)	C6B—H6B	0.9300
C4B—C5B	1.390 (12)	C7A—H7A	0.9300
C5A—C6A	1.390 (9)	C7B—H7B	0.9300
C5B—C6B	1.390 (12)	C8A—H8A	0.9300
C6A—C7A	1.391 (10)	C8B—H8B	0.9300
C6B—C7B	1.391 (13)	C10A—H10A	0.9300
C7A—C8A	1.390 (9)	C10B—H10B	0.9300
C7B—C8B	1.390 (12)	C11A—H11A	0.9300
C9A—C14A	1.390 (12)	C11B—H11B	0.9300
C9A—C10A	1.390 (13)	C12A—H12A	0.9300
C9B—C14B	1.390 (15)	C12B—H12B	0.9300
C9B—C10B	1.390 (16)	C13A—H13A	0.9300
C10A—C11A	1.390 (13)	C13B—H13B	0.9300
C10B—C11B	1.391 (14)	C14A—H14A	0.9300
C11A—C12A	1.390 (9)	C14B—H14B	0.9300
C11B—C12B	1.390 (11)	C16A—H16A	0.9300
C12A—C13A	1.390 (10)	C16B—H16B	0.9300
C12B—C13B	1.390 (14)	C17A—H17A	0.9300
C13A—C14A	1.391 (12)	C17B—H17B	0.9300
C13B—C14B	1.391 (16)	C18A—H18A	0.9300
C15A—C16A	1.391 (10)	C18B—H18B	0.9300
C15A—C20A	1.390 (10)	C19A—H19A	0.9300
C15B—C20B	1.390 (12)	C19B—H19B	0.9300
C15B—C16B	1.390 (13)	C20A—H20A	0.9300
C16A—C17A	1.389 (10)	C20B—H20B	0.9300
			
S1—Sn1—C3A	107.67 (18)	O1—C2—H2B	109.00
S1—Sn1—C9A	98.9 (3)	O1—C2—H2C	109.00
S1—Sn1—C15A	108.02 (19)	H2A—C2—H2B	109.00
S1—Sn1—C3B	107.3 (2)	H2A—C2—H2C	109.00
S1—Sn1—C9B	97.5 (3)	H2B—C2—H2C	109.00
S1—Sn1—C15B	110.3 (3)	C3A—C4A—H4A	120.00
C3A—Sn1—C9A	112.9 (3)	C5A—C4A—H4A	120.00
C3A—Sn1—C15A	115.1 (3)	C5B—C4B—H4B	120.00
C9A—Sn1—C15A	112.7 (3)	C3B—C4B—H4B	120.00
C3B—Sn1—C9B	113.0 (4)	C6A—C5A—H5A	120.00
C3B—Sn1—C15B	115.9 (4)	C4A—C5A—H5A	120.00
C9B—Sn1—C15B	111.1 (4)	C6B—C5B—H5B	120.00
Sn1—S1—C1	101.02 (16)	C4B—C5B—H5B	120.00
C1—O1—C2	119.0 (4)	C7A—C6A—H6A	120.00
S1—C1—S2	121.5 (3)	C5A—C6A—H6A	120.00
S1—C1—O1	111.7 (3)	C7B—C6B—H6B	120.00
S2—C1—O1	126.8 (4)	C5B—C6B—H6B	120.00
Sn1—C3A—C4A	118.0 (4)	C8A—C7A—H7A	120.00
Sn1—C3A—C8A	121.9 (6)	C6A—C7A—H7A	120.00
C4A—C3A—C8A	120.0 (6)	C6B—C7B—H7B	120.00
Sn1—C3B—C8B	117.4 (7)	C8B—C7B—H7B	120.00
C4B—C3B—C8B	120.0 (7)	C7A—C8A—H8A	120.00
Sn1—C3B—C4B	122.5 (7)	C3A—C8A—H8A	120.00
C3A—C4A—C5A	120.0 (6)	C7B—C8B—H8B	120.00
C3B—C4B—C5B	120.0 (9)	C3B—C8B—H8B	120.00
C4A—C5A—C6A	120.0 (7)	C11A—C10A—H10A	120.00
C4B—C5B—C6B	120.0 (9)	C9A—C10A—H10A	120.00
C5A—C6A—C7A	120.0 (5)	C11B—C10B—H10B	120.00
C5B—C6B—C7B	120.0 (7)	C9B—C10B—H10B	120.00
C6A—C7A—C8A	120.0 (6)	C12A—C11A—H11A	120.00
C6B—C7B—C8B	120.0 (8)	C10A—C11A—H11A	120.00
C3A—C8A—C7A	120.0 (7)	C12B—C11B—H11B	120.00
C3B—C8B—C7B	120.0 (8)	C10B—C11B—H11B	120.00
Sn1—C9A—C10A	116.9 (6)	C11A—C12A—H12A	120.00
Sn1—C9A—C14A	122.9 (7)	C13A—C12A—H12A	120.00
C10A—C9A—C14A	120.1 (9)	C11B—C12B—H12B	120.00
C10B—C9B—C14B	120.0 (10)	C13B—C12B—H12B	120.00
Sn1—C9B—C10B	126.1 (7)	C14A—C13A—H13A	120.00
Sn1—C9B—C14B	113.8 (9)	C12A—C13A—H13A	120.00
C9A—C10A—C11A	120.0 (7)	C14B—C13B—H13B	120.00
C9B—C10B—C11B	120.0 (8)	C12B—C13B—H13B	120.00
C10A—C11A—C12A	120.0 (7)	C13A—C14A—H14A	120.00
C10B—C11B—C12B	120.0 (9)	C9A—C14A—H14A	120.00
C11A—C12A—C13A	120.0 (7)	C13B—C14B—H14B	120.00
C11B—C12B—C13B	120.0 (8)	C9B—C14B—H14B	120.00
C12A—C13A—C14A	120.0 (6)	C17A—C16A—H16A	120.00
C12B—C13B—C14B	120.0 (8)	C15A—C16A—H16A	120.00
C9A—C14A—C13A	119.9 (8)	C17B—C16B—H16B	120.00
C9B—C14B—C13B	120.0 (11)	C15B—C16B—H16B	120.00
Sn1—C15A—C16A	116.8 (5)	C16A—C17A—H17A	120.00
C16A—C15A—C20A	120.0 (7)	C18A—C17A—H17A	120.00
Sn1—C15A—C20A	123.2 (5)	C18B—C17B—H17B	120.00
Sn1—C15B—C20B	115.4 (7)	C16B—C17B—H17B	120.00
C16B—C15B—C20B	120.0 (9)	C19A—C18A—H18A	120.00
Sn1—C15B—C16B	124.6 (7)	C17A—C18A—H18A	120.00
C15A—C16A—C17A	120.0 (6)	C19B—C18B—H18B	120.00
C15B—C16B—C17B	120.0 (8)	C17B—C18B—H18B	120.00
C16A—C17A—C18A	120.0 (6)	C20A—C19A—H19A	120.00
C16B—C17B—C18B	120.0 (8)	C18A—C19A—H19A	120.00
C17A—C18A—C19A	120.0 (6)	C20B—C19B—H19B	120.00
C17B—C18B—C19B	120.0 (9)	C18B—C19B—H19B	120.00
C18A—C19A—C20A	120.0 (6)	C19A—C20A—H20A	120.00
C18B—C19B—C20B	120.0 (7)	C15A—C20A—H20A	120.00
C15A—C20A—C19A	120.1 (5)	C15B—C20B—H20B	120.00
C15B—C20B—C19B	120.0 (8)	C19B—C20B—H20B	120.00
O1—C2—H2A	109.00		
			
C3A—Sn1—S1—C1	46.2 (3)	Sn1—C3A—C4A—C5A	176.3 (6)
C9A—Sn1—S1—C1	163.8 (3)	C8A—C3A—C4A—C5A	0.0 (12)
C15A—Sn1—S1—C1	−78.7 (3)	Sn1—C3A—C8A—C7A	−176.2 (6)
S1—Sn1—C3A—C4A	−155.3 (6)	C4A—C3A—C8A—C7A	−0.1 (12)
S1—Sn1—C3A—C8A	20.9 (7)	C3A—C4A—C5A—C6A	0.1 (12)
C9A—Sn1—C3A—C4A	96.6 (7)	C4A—C5A—C6A—C7A	−0.1 (11)
C9A—Sn1—C3A—C8A	−87.2 (7)	C5A—C6A—C7A—C8A	0.0 (11)
C15A—Sn1—C3A—C4A	−34.8 (7)	C6A—C7A—C8A—C3A	0.1 (11)
C15A—Sn1—C3A—C8A	141.4 (6)	Sn1—C9A—C10A—C11A	177.4 (6)
S1—Sn1—C9A—C10A	−104.6 (7)	C14A—C9A—C10A—C11A	0.0 (14)
S1—Sn1—C9A—C14A	72.7 (8)	Sn1—C9A—C14A—C13A	−177.3 (6)
C3A—Sn1—C9A—C10A	8.9 (8)	C10A—C9A—C14A—C13A	−0.1 (13)
C3A—Sn1—C9A—C14A	−173.8 (7)	C9A—C10A—C11A—C12A	0.0 (13)
C15A—Sn1—C9A—C10A	141.5 (7)	C10A—C11A—C12A—C13A	0.0 (11)
C15A—Sn1—C9A—C14A	−41.2 (9)	C11A—C12A—C13A—C14A	0.0 (11)
S1—Sn1—C15A—C16A	−3.9 (6)	C12A—C13A—C14A—C9A	0.1 (12)
S1—Sn1—C15A—C20A	175.7 (5)	Sn1—C15A—C16A—C17A	179.7 (5)
C3A—Sn1—C15A—C16A	−124.3 (5)	C20A—C15A—C16A—C17A	0.0 (10)
C3A—Sn1—C15A—C20A	55.4 (7)	Sn1—C15A—C20A—C19A	−179.7 (5)
C9A—Sn1—C15A—C16A	104.2 (6)	C16A—C15A—C20A—C19A	−0.1 (11)
C9A—Sn1—C15A—C20A	−76.1 (7)	C15A—C16A—C17A—C18A	0.0 (10)
Sn1—S1—C1—S2	−161.2 (3)	C16A—C17A—C18A—C19A	0.0 (10)
Sn1—S1—C1—O1	18.0 (4)	C17A—C18A—C19A—C20A	0.0 (10)
C2—O1—C1—S1	−174.0 (4)	C18A—C19A—C20A—C15A	0.1 (10)
C2—O1—C1—S2	5.1 (7)		
